# Association of circulating irisin and cardiopulmonary exercise capacity in healthy volunteers: results of the Study of Health in Pomerania

**DOI:** 10.1186/s12890-015-0035-x

**Published:** 2015-04-22

**Authors:** Nils Kerstholt, Ralf Ewert, Matthias Nauck, Thomas Spielhagen, Tom Bollmann, Beate Stubbe, Stephan B Felix, Henri Wallaschofski, Sven Gläser, Nele Friedrich

**Affiliations:** Institute of Clinical Chemistry and Laboratory Medicine, University Medicine Greifswald, Ferdinand-Sauerbruch-Straße, 17475 Greifswald, Germany; Department of Internal Medicine B – Cardiology, Intensive Care, Pulmonary Medicine and Infectious Diseases, University Medicine Greifswald, Ferdinand-Sauerbruch-Straße, 17475 Greifswald, Germany; DZHK (German Centre for Cardiovascular Research), Greifswald partner site, Greifswald, Germany

**Keywords:** Irisin, Cardiopulmonary exercise capacity, Ergometer, SHIP-TREND

## Abstract

**Background:**

Irisin, a recently discovered myokine, is assumed to be secreted by muscle cells in response to exercise and is involved in the regulation of energy metabolism by browning white adipose tissue cells. However, due to the fact that previous studies revealed conflicting results concerning the association between irisin and exercise, the aim of the present study was to investigate the potential relationship between irisin and exercise capacity in a population-based setting.

**Methods:**

From the population-based Study of Health in Pomerania (SHIP-TREND) 334 men and 406 women with irisin measurements were selected and a standardised symptom limited cardiopulmonary exercise test was used. Exercise capacity was quantified by oxygen uptake at anaerobic threshold (VO_2_@AT), peak exercise (peakVO_2_) and maximum power output at peak exertion. In addition, the oxygen pulse was assessed. ANOVA and multivariable linear regression analyses were performed stratified by sex and adjusted for age, weight, height and smoking.

**Results:**

In men, we observed inverse associations between irisin serum concentration and exercise capacity assessed by peakVO_2_ and maximum power output. In contrast, in women a trend towards a positive relationship between irisin and peakVO_2_ was detected, whereas none of the other parameters showed significant associations with irisin.

**Conclusion:**

Based on a large population sample, our results did not confirm the previous reported positive linkage between exercise and irisin. Thus the relationship needs further investigation in particular with respect to sex differences.

**Electronic supplementary material:**

The online version of this article (doi:10.1186/s12890-015-0035-x) contains supplementary material, which is available to authorized users.

## Background

Myokines have an important role in the human body, especially in coordinating the different organs and tissues such as the adipose tissue, liver, pancreas, bones, brain and muscle tissue; also influencing human homoeostasis [1-3]. In 2012, Boström et al. [4] discovered a new myokine called irisin, which is secreted by muscle tissue and is transcribed from the FNDC5 (fibronectin type III domain-containing protein 5) gene. Irisin is assumed to affect white adipose tissue cells by browning them, as well as increasing energy expenditure. Furthermore, irisin seems to promote increased oxygen consumption and energy supply [5]. These effects suggest that irisin might be the source of a potential therapeutic approach to treating obesity and diabetes mellitus, two major diseases in industrialized countries [6-11].

According to Norheim et al. [12] irisin was first known as a peroxisome proliferator-activated receptor γ co-activator-1α (PGC-1α) dependent myokine with browning effects in murine adipocytes. In 2013, researchers [12] found a positive correlation with human muscle cells among 26 diabetic and non-diabetic subjects aged 40–65 years. The study was able to show an increase in mRNA for PGC1 α and FNDC5 after 12 weeks of physical training in both control and pre-diabetes human subjects. Furthermore, long-term training led to an overall reduction in circulating irisin levels whereas an increase in irisin were only found after acute exercise. A similar trend was shown among 12 subjects undergoing treadmill exercise [13]. A temporary increase in irisin levels during the first hour of exercise was revealed, whereas after 90 minutes of exercise irisin levels were no longer elevated. In contrast to these studies, several investigations [14-17] failed to confirm these findings. One study published by Hecksteden et al. [17] based on an experiment including 102 participants aged 30–60 years exposed to aerobic endurance training and strength endurance training. This study and three others [14-16] did not confirm an exercise related increase in irisin levels.

However, as Hofmann et al. [8] and Novelle et al. [10] have already stated, the results on “the regulation of Irisin by different types of exercise are partly conflicting and effects were only shown in highly selective patient populations so far” and therefore “it is still necessary to deepen in several aspects in order to clarify its full potential as a meaningful drug target in human disease states”.

Therefore the aim of the present study is to investigate the association of irisin serum concentration with exercise capacity in a subsample of the Study of Health in Pomerania Trend (SHIP-TREND).

## Methods

SHIP-TREND is a population-based study in Northeast Germany including the cities of Greifswald, Stralsund, Anklam and 29 surrounding communities [18]. A representative sample of 8016 adults aged 20 to 79 years was randomly selected from population registries into 24 age- and sex-specific strata. In total 4420 subjects participated in the baseline examination of SHIP-TREND between September 2008 and September 2012. The study follows the recommendations of the Declaration of Helsinki and was approved by the ethics committee of the University of Greifswald. All participants gave written informed consent.

Measurements of irisin levels were available for a subsample of 1000 subjects. Of these, 260 subjects were excluded due to the presence of at least one of the following conditions: no participation in cardiopulmonary exercise testing (CPET), pulmonary function testing performed later than 100 days after core examination, estimated glomerular filtration rate <30 mL/min/1.73 m^2^ as well as conditions related to cardiovascular diseases including pacemaker, aortic or mitral valve stenosis, intake of digitalis glycosides [anatomical therapeutic chemical (ATC) code C01AA] or selective calcium channel blockers with direct cardiac effects (ATC code C08D). Those with missing values for confounding factors were also excluded. The final study population thus comprised 740 individuals (334 men, 406 women).

### Measurements

Information on age, gender, socio-demographic characteristics and medical histories were obtained by computer-aided personal interviews. Smoking status and physical activity were assessed by self-report. Individuals who participated in physical training during summer or winter for at least one hour a week were classified as being physically active. Waist circumference (WC) was measured to the nearest 0.1 cm using an inelastic tape midway between the lower rib margin and the iliac crest in the horizontal plane, with the subject standing comfortably with weight distributed evenly on both feet. The measurement was taken at the level of the narrowest part of the waist. All anthropometric measurements were taken in accordance with World Health Organization standards.

During the core examination of all participants, fasting blood samples were drawn from the cubital vein in the supine position and serum aliquots were prepared for immediate analysis and for storage at −80°C. Irisin levels were measured by a competitive enzyme-linked immunosorbent assay (Adipogen AG, Liestal, Switzerland) with a limit of detection of 0.001 μg/ml. During the course of the study the coefficient of variation was 11.57%.

### Exercise testing and gas exchange variables

A symptom-limited exercise test using a calibrated electromagnetically braked cycle ergometer with an electrical seat height adjustment (Ergoselect 100, Ergoline, Germany) was performed according to a modified Jones protocol (stepwise increase in work load of 16 Watts/minute, starting with unloaded cycling plus the ergometer related permanent load) [19]. Gas exchange and ventilatory variables were analysed breath by breath averaged over 10-second intervals using a computer-based system; study details are given elsewhere [20]. In the absence of chest pain and ECG abnormalities, all tests were continued as symptom-limited (volitional exertion, dyspnoea or fatigue). Prior to the test, patients were encouraged to reach maximal exhaustion, while during exercise no further motivational interventions were made. All tests were performed at room air according to current guidelines for exercise testing, with continuous monitoring of ECG, blood pressure and oxygen saturation [21,22].

Peak oxygen uptake (peakVO_2_) was defined as the highest 10-second average of VO_2_ in the last minute of exercise. The oxygen uptake at anaerobic threshold (VO_2_@AT) was determined according to Wasserman et al. [23]. The determination of anaerobic threshold (AT) was based on three methods: non-invasive determination by gas exchange analysis by investigating the relation of VO_2_ to VCO_2_ (V-slope method), end-tidal gas concentrations over time and ventilatory equivalents for oxygen and carbon dioxide over time [23]. At least two of the three methods had to be compliant. Maximal power output was characterised as the highest reached power in Watts (W) which was maintained for at least 20 seconds on the bicycle ergometer during exercise. All investigated parameters have been shown to be reliably reproducible [24]. Exercise duration was investigated as from the start of exercise (without resting period) up to its termination.

In the present study CPET was performed up to 100 days after core investigation (blood sampling). The median time-lag was 28 days (25^th^ percentile 9 days; 75^th^ percentile 48 days). All analyses were also performed in a subpopulation of 402 subjects with a maximal time-lag of one month [median 11 days (25^th^ percentile 3 days; 75^th^ percentile 21 days)].

### Statistical analysis

Continuous data are expressed as median (25^th^ percentile; 75^th^ percentile). Nominal data are expressed as percentages. For bivariate analyses the Kruskal-Wallis test (continuous data) or *χ*^2^-test (nominal data) were used to compare men and women, as well as participants and non-participants. Analysis of variance (ANOVA) was carried out to calculate adjusted means for CPET parameters in groups (categorisation according to sex- and months-specific tertiles). Multivariable linear regression models were separately performed in men and women to estimate the independent associations of irisin as a continuous variable with CPET parameters. To detect possible nonlinear associations, for both analyses models with restricted cubic splines with 3 knots pre-specified located at the 5^th^, 50^th^, and 95^th^ percentile as recommended by Stone and Koo [25] were compared by a likelihood ratio test to the fit of the linear model. The full models were adjusted for age, body-mass index, smoking, time between core examination and CPET and months of core examination. Furthermore, in linear regression analyses glucose and total cholesterol levels were tested as further potential confounders. Sensitivity analyses were performed with either the exclusion of 1) all subjects with chronic obstructive pulmonary disease (COPD), defined as FEV1/FVC <0.7 or intake of drugs for obstructive airway diseases (ATC code R03), or 2) all subjects with a time-lag between blood sampling and CPET of more than one month. A value of p < 0.05 was considered statistically significant. Statistical analyses were performed with SAS 9.3 (SAS Institute Inc., Cary, NC, USA).

## Results

**Figure 1:**
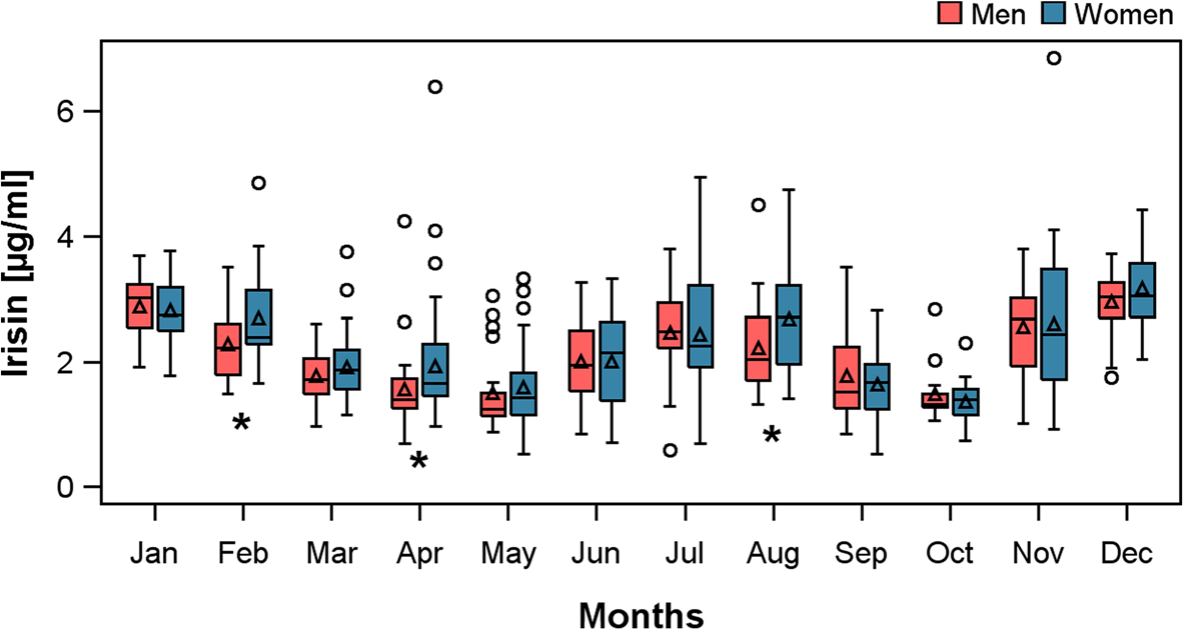
Boxplots of irisin levels separately for men (blue) and women (red) by month. * p < 0.05 for comparison between men and women.

Men and women were compared according to general characteristics (Table 1). Women were more often never smokers and had a lower WC and body-mass index than men. Furthermore, women were less often affected by hypertension and had lower blood glucose but higher total cholesterol levels when compared to men. With respect to CPET parameters, men had higher levels of peakVO_2_, VO_2_@AT, oxygen pulse and a higher power output at peak exercise than women. Regarding irisin no overall sex differences were apparent. However, our data showed a strong annual rhythm of irisin levels. As displayed in Figure 1, irisin showed peak values in the winter (December – February) and summer months (July – August) compared to the remaining months. Even if men and women showed similar rhythms, women exhibited significant higher irisin levels in selected months. Compared to non-participants, the participants were more often women, never smokers, more physically active, had a lower body-mass index and glucose levels and were less often affected by hypertension.

**Table 1:** **Descriptive statistics of the study sample**

	**Non-participants (N = 3658)**	**Study population**				
		**Complete (N = 740)**	**p** ^**†**^	**Men (N = 322)**	**Women (N = 418)**	**p** ^**‡**^
Age (years)*	53 (39.5; 65)	51 (41; 61)	<0.01	50 (39; 61)	52 (41; 61)	0.54
Men (%)	49.2	45.1	0.04	-	-	
Smoking (%)			<0.01			<0.01
never smokers	35.3	42.4		32.0	51.0	
former smokers	36.4	37.8		48.5	29.1	
current smokers	28.4	19.7		19.5	19.9	
Physical activity (%)	67.9	75.0	<0.01	73.9	75.8	0.55
Waist circumference (cm)	91.4 (80.9; 102.0)	87.1 (78.5; 97.0)	<0.01	94.0 (86.5; 102.5)	81.1 (74.0; 90.0)	<0.01
Body-mass-index (kg/m^2^)	27.7 (24.5; 31.3)	26.8 (24.1; 30.0)	<0.01	27.8 (25.0; 30.3)	26.1 (23.2; 29.5)	<0.01
Glucose (mmol/l)	5.4 (5.0; 6.0)	5.3 (4.9; 5.7)	<0.01	5.4 (5.1; 5.9)	5.2 (4.9; 5.6)	<0.01
Total cholesterol (mmol/l)	5.4 (4.6; 6.1)	5.4 (4.7; 6.2)	0.11	5.3 (4.6; 6.1)	5.5 (4.9; 6.2)	0.02
Hypertension (%)	50.1	38.7	<0.01	43.1	35.1	0.03
Irisin (μg/ml)	-	-	-	1.97 (1.47; 2.70)	2.01 (1.48; 2.71)	0.69
peak VO_2_ (ml/min)	-	-	-	2600 (2183; 3000)	1611 (1400; 1925)	<0.01
VO_2_@AT (ml/min)	-	-	-	1200 (1000; 1400)	850 (800; 1000)	<0.01
Oxygen pulse (ml)	-	-	-	16.1 (14.4; 18.1)	10.9 (9.3; 12.2)	<0.01
Maximum power output (watt)	-	-	-	196 (180; 228)	132 (116; 148)	<0.01

Peak VO_2_ = highest 10-second average of VO_2_ in the last minute of exercise; VO_2_@AT = VO_2_ at lactate threshold. Continuous data are expressed as median (25^th^ and 75^th^ percentiles; nominal data are given as percentages. *χ* ^2^-test (nominal data) or Kruskal-Wallis test (interval data) were used to compare non-participants with participants (^†^) or participating men and women (^**‡**^). *Age at core examination.

**Figure 2:**
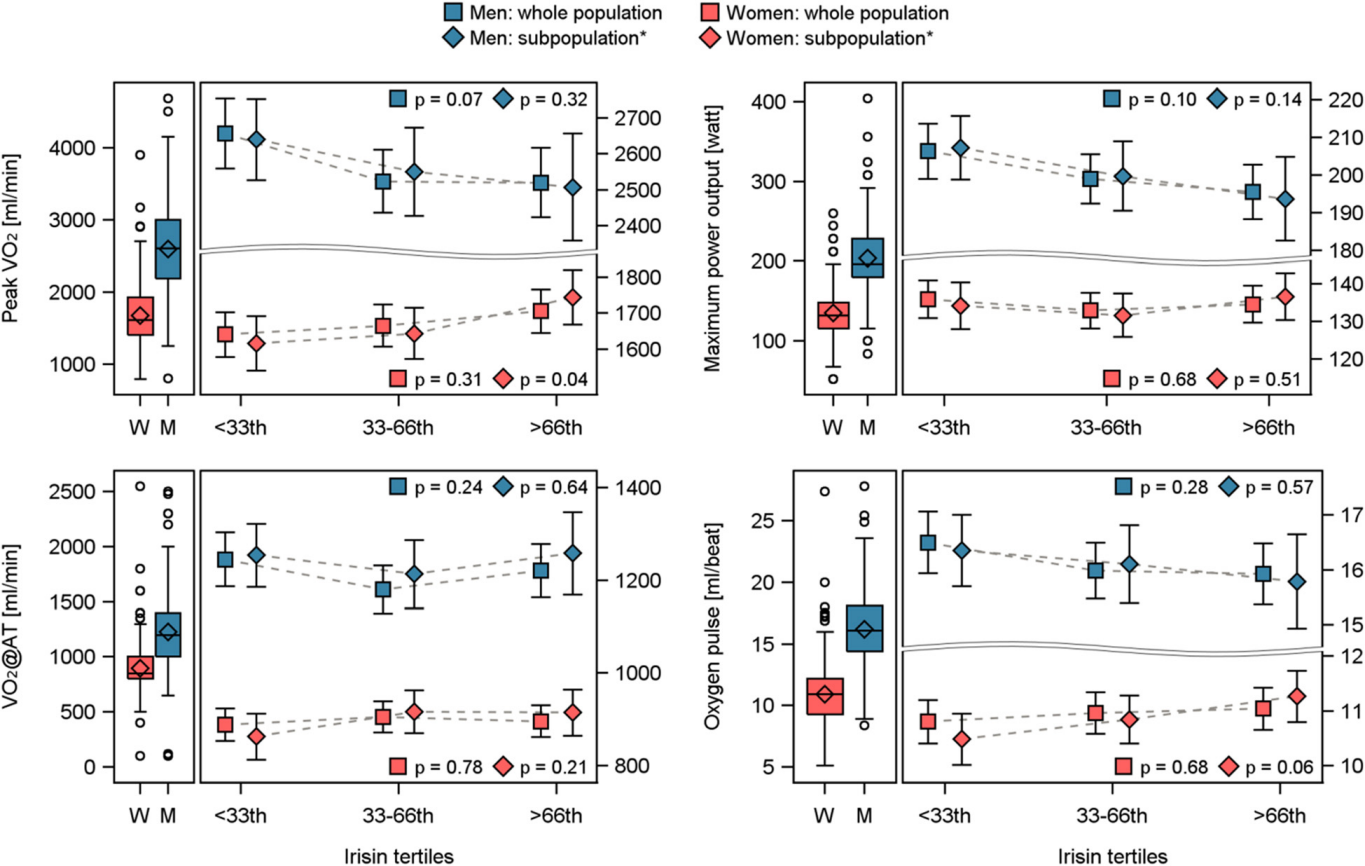
Estimated mean level of highest 10-second average of VO_2_ in the last minute of exercise (peak VO_2_), VO_2_ at lactate threshold (V0_2_@AT), maximum power output and oxygen pulse with 95% confidence interval by sex- and month-specific tertiles of irisin. Analysis of variance was adjusted for age, body-mass index, smoking and time between core examination and pulmonary function testing. *The whole population as well as only subjects with a maximal time-lag between blood sampling and pulmonary function testing of one month.

In the whole population, ANOVA (Figure 2) revealed trends towards inverse associations between categories of irisin and peakVO_2_ as well as maximum power output among men. The estimated mean level of peakVO_2_ or maximum power output was 138 ml/min or 11 watts higher in the first [peakVO_2_ (ml/min): 2656 (95% confidence interval (CI) 2566, 2747); maximum power output (watt): 207 (95%-CI 200, 214)] compared to the third tertile [peakVO_2_: 2518 (95%-CI 2428, 2518); maximum power output: 196 (95%-CI 189, 202)], respectively. The associations became weaker after the exclusion of men with a time-lag between blood sampling and CPET of more than one month (Figure 2). However, still higher estimated mean levels of peakVO_2_ [1^st^ tertile: 2639 (95%-CI 2527, 2752); 3^rd^ tertile: 2508 (95%-CI 2359, 2656)] or maximum power output [1^st^ tertile: 207 (95%-CI 199, 216); 3^rd^ tertile: 193 (95%-CI 183, 205)] could be observed in the first compared to the third irisin tertile. In the subgroup of women with a maximal time-lag of one month, categorical analyses suggest a positive association between irisin tertiles and oxygen pulse [1^st^ tertile: 10.5 (95%-CI 10.0, 11.0); 3^rd^ tertile: 11.3 (95%-CI 10.8, 11.7)]. No association between irisin and VO_2_@AT or oxygen pulse was found in either sex.

**Figure 3:**
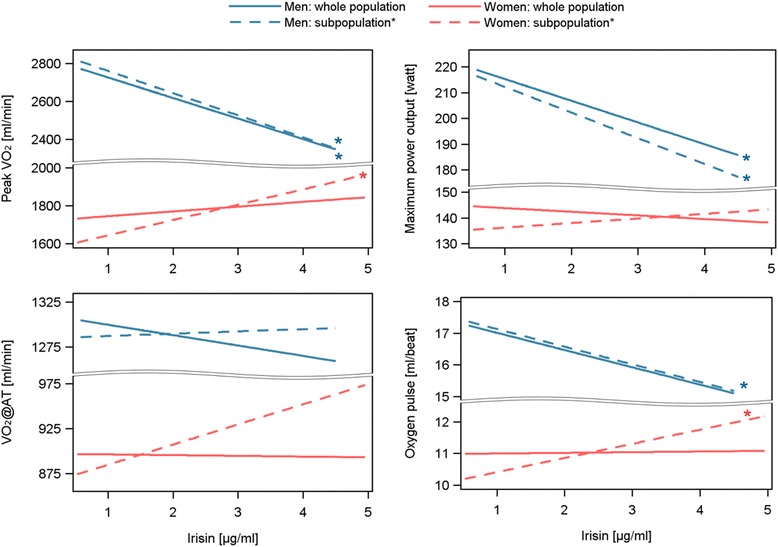
Predicted mean of highest 10-second average of VO2 in the last minute of exercise (peak VO2), VO2 at lactate threshold (V02@AT), maximum power output and oxygen pulse depending on irisin levels calculated by linear regression analyses for men (blue) and women (red). Models were adjusted for 1) age, body-mass index, smoking, glucose, total cholesterol levels as well as time between core examination and pulmonary function testing and months of core examination. *The whole population (solid lines) as well as only subjects with a maximal time-lag between blood sampling and pulmonary function testing of one month (dashed lines).

Fully adjusted linear regression analyses confirmed the inverse association between irisin and peakVO_2_ as well as maximum power output (Figure 3 and Table 2) and additionally revealed an inverse relationship with oxygen pulse in men. These findings were independent of the study population used. Furthermore, among women, after excluding those with a large time-lag between blood sampling and CPET, significant positive associations between continuous irisin levels and peakVO_2_ or oxygen pulse became apparent in a fully adjusted model. No further significant associations were found in women. The additional adjustment for self-reported physical activity did not change the detected findings (Additional file 1: Table S1). The exclusion of subjects with COPD also confirmed the reported findings of the linear regression analyses even though some of the relationships were short of being statistically significant (Additional file 1: Table S2). One reason for the weaker associations might be the loss of power.

**Table 2:** **Association between irisin and CPET parameters depending on different adjustment sets**

	**peakVO**_**2**_		**VO**_**2**_ **@AT**		**Oxygen pulse**		**Maximum power output**	
	**ß coefficient (Stderr)**	**p**	**ß coefficient (Stderr)**	**p**	**ß coefficient (Stderr)**	**p**	**ß coefficient (Stderr)**	**p**
***Whole population***								
**Men (n = 322)**								
adjusted for age, BMI, smoking	−97.43 (43.139)	0.02	−6.388 (26.278)	0.81	−0.463 (0.249)	0.06	−7.656 (3.258)	0.02
adjusted for age, BMI, smoking, glucose, total cholesterol	−108.2 (43.752)	0.01	−11.48 (26.618)	0.67	−0.544 (0.252)	0.03	−8.434 (3.305)	0.01
**Women (n = 418)**								
adjusted for age, BMI, smoking	27.580 (23.166)	0.23	0.981 (13.347)	0.94	0.054 (0.149)	0.72	−1.296 (1.872)	0.49
adjusted for age, BMI, smoking, glucose, total cholesterol	25.103 (23.337)	0.28	−0.805 (13.436)	0.95	0.021 (0.150)	0.89	−1.410 (1.887)	0.46
***Subpopulation of subjects with a maximal time-lag between blood sampling and pulmonary function testing of one month***								
**Men (n = 177)**								
adjusted for age, BMI, smoking	−96.07 (58.396)	0.10	4.926 (35.355)	0.89	−0.442 (0.335)	0.19	−8.720 (4.413)	0.05
adjusted for age, BMI, smoking, glucose, total cholesterol	−116.9 (60.121)	0.05	2.569 (36.563)	0.94	−0.557 (0.342)	0.11	−9.979 (4.557)	0.03
**Women (n = 225)**								
adjusted for age, BMI, smoking	80.507 (27.836)	<.01	24.328 (18.694)	0.19	0.455 (0.177)	0.01	1.839 (2.302)	0.43
adjusted for age, BMI, smoking, glucose, total cholesterol	80.805 (28.106)	<.01	22.367 (18.856)	0.24	0.446 (0.179)	0.01	1.775 (2.326)	0.45

BMI = body-mass index; peakVO_2_ = highest 10-second average of VO_2_ in the last minute of exercise; VO_2_@AT = VO_2_ at anaerobic threshold. All models were further adjusted for time between core examination and pulmonary function testing and month of core examination.

## Discussion

In the present study inverse associations between irisin and exercise capacity assessed by peakVO_2_, oxygen pulse and maximum power output were found among men. Furthermore, in a subgroup of women with a time-lag between blood sampling and CPET of less than one month, however, positive relationships between irisin and peakVO_2_ as well as oxygen pulse were detected.

Irisin is a recently discovered PGC1α dependent myokine. Originally PGC1α has been described as a co-activator of biological mechanisms linked with energy metabolism. In concordance with our results toward a positive association between irisin and peakVO_2_ in women, Boström and colleagues [4] reported an exercise-related increase in expression of FNDC5 and consequently increased irisin levels in mice after three weeks of free wheel running. The authors also confirmed their findings among eight healthy but obese participants by showing a significant increase in irisin levels after 10 weeks of endurance training. However, the study population was rather small to drawn conclusions from these results. Recently published studies [12,13,26] further revealed an acute increase in irisin levels after 30 min, 45 min or 54 min of endurance exercise. However, after 90 min or 8 weeks of training irisin levels were no longer elevated [13] and even more interesting, after 12 weeks of training a decrease in irisin levels was observed in 13 healthy and 13 pre-diabetic participants [12]. The long-term reduction in irisin levels is in concordance with present detected inverse relationships between irisin and peakVO_2_ or maximum power output among men. However, these studies are contradicted by a study [27] among 163 Japanese men also using cardiopulmonary exercise parameters which did not revealed any correlation between peak VO_2_ and irisin levels. Reasons for the different findings might be the different sample size as well as different methods used to measure irisin levels.

In general, there is a broad range of studies [15-17] that indicate no relationship between exercise and irisin levels. An investigation among 102 participants who took part in either a strength or aerobic endurance training program for 26 weeks did not show any exercise-induced increase in irisin levels [17]. Further studies conducted among healthy [15,16] and obese subjects [14] as well as among haemodialysis patients [15] and patients with anorexia nervosa [28], also showed no significant alterations in irisin levels after aerobic exercise, resistance exercise or/and endurance exercise. The majority of these studies were conducted in experimental settings and included less than 100 subjects, therefore the present results based on over 700 subjects might further contribute to the ongoing discussion of the physiological and pathophysiological actions of irisin.

Most of the studies analysed both men and women at the same time, which might be a further reason for the inconsistent results. One previous study [29], also performed sex-specific analyses and firstly revealed a sexual dimorphic response to circulating irisin levels. Whereas in men, a negative association between exercise and circulating irisin levels was found, a positive association became apparent among women. These findings support our results regarding exercise parameters in men and women. The authors suggest differences in transcription levels of FNDC5, body composition or sexual hormones as possible explanations [29]. However, in the present study population, no correlation between irisin levels and either anthropometric marker, including body-mass index or visceral and subcutaneous fat measured based on MRI, or sexual hormones including total testosterone, androstenedione or estradiol became apparent. Therefore, further studies are needed to elucidate the mechanism underlying the sex differences regarding the relationship between irisin levels and exercise capacity.

The majority of the above-mentioned studies examined the direct association between various types of exercise and irisin levels. One possible reason for the discrepancies between the previous studies and ours might be that the present study represents a cross-sectional investigation of the association between physical performance measured by cardiopulmonary exercise testing and irisin levels and is therefore more likely to reflect the physical and training status when the exercise test was performed. Our study did not investigate the effects of exercise itself on irisin levels. Furthermore, the conflicting findings might be caused by different study settings which lead to different distributions of confounding factors such as age, body composition, cholesterol levels or fitness status. A recent study [26] has dealt with these confounding factors and clearly demonstrated that irisin levels were positively correlated with biceps circumference (as a marker of muscle mass herein), body-mass index, glucose ghrelin and insulin-like growth factor I levels and, on the other side, negatively correlated with age, insulin, cholesterol and adiponectin levels. In general, besides several influencing factors causing fluctuations in irisin levels, the origin of irisin is still not fully understood. A previous study [30] found that irisin is not only a myokine but also an adipokine and other studies [6,26] detected irisin in a great variety of tissues ranging from pericardium, rectum and heart to kidney, liver, lung and adipose tissue. Thus it is very questionable if irisin is actually mainly secreted by muscle tissue or just accidently secreted in this tissue in response to exercise. Therefore, further investigations are needed to clarify the potential physiological association between irisin and exercise as well as obesity.

Additionally, we came across something unexpected and new in the history of irisin: an annual rhythm of irisin levels. According to the collected data irisin levels show two peaks, one during the summer and the other during the winter period. A possible explanation might be a variation in physical activity during the year. In summer months, people are more likely to be active outside, to do sports and to walk around. This might lead to an exercise-related increase in irisin levels. During the winter period, to compensate for lower outside temperature the overall exercise rate might be higher related to the rise in energy expenditure and shivering-linked thermogenesis. Another speculative reason might be the higher percentage of adipose tissue present during the winter months. Currently irisin has been found to be an adipokine which is secreted by white adipose tissue and especially by subcutaneous adipose tissue [30].

A further reason might be heterogeneity between subjects examined in different months. Even if we found no significance differences for general characteristics e.g. age, smoking, body-mass index, glucose levels or proportion of hypertension (data not shown) between subjects examined in different months, further studies are needed to clarify the detected annual rhythm of irisin levels.

The strengths of the present study are the large population of over 700 participants and the accurate assessment of confounding factors and exercise parameters based on standardised protocols. Furthermore, we firstly investigated the possible sex-specific difference in the association between exercise and irisin levels in a large-scale population sample. However, there are also limitations. With respect to the interpretation of the results, there might be a problem regarding linking the oxygen uptake directly to the exercise taken. We assume that our method to assess physical capacity might have a large influence on the results. Furthermore, factors like menopause status which might influence the muscle status of women were not considered due to the lack of reliable data.

## Conclusion

In conclusion, we detected inverse associations between irisin and exercise capacity assessed by peakVO_2_ and maximum power output among men and a trend toward a positive relationship between irisin and peakVO_2_ among women, suggesting possible sex differences.

## Availability of supporting data

SHIP data are publically available for scientific and quality control purposes. Data usage can be applied for via www.community-medicine.de.

## Additional file

Additional file 1: Table S1. Association between irisin and CPET parameters additionally adjusted for self-reported physical activity. **Table S2.** Association between irisin and CPET parameters depending on different adjustment sets in a COPD free population.

## Abbreviations

ANOVA: Analysis of variance
ATC: Anatomical therapeutic chemical
CI: Confidence interval
CPET: Cardiopulmonary exercise testing
ECG: Electrocardiography
FNDC5: Fibronectin type III domain-containing protein 5
peakVO_2_: Peak oxygen uptake
PGC-1α: Peroxisome proliferator-activated receptor γ co-activator-1α
SHIP-TREND: Study of health in West Pomerania trend
VO_2_@AT: Oxygen uptake at anaerobic threshold
WC: Waist circumference
