# Supplementary material for: Association of circulating irisin and cardiopulmonary exercise capacity in healthy volunteers: results of the Study of Health in Pomerania
Source: BMC Pulm Med. 2015 Apr 22;15:41. doi: 10.1186/s12890-015-0035-x (PMC4416285; doi:10.1186/s12890-015-0035-x)
Supplement: Additional file 1: Table S1. — Association between irisin and CPET parameters additionally adjusted for self-reported physical activity. Table S2. Association between irisin and CPET parameters depending on different adjustment sets in a COPD free population. [file 12890_2015_35_MOESM1_ESM.docx]

**Additional file 1**

**Table S1.** Association between irisin and CPET parameters additionally adjusted for self-reported physical activity.

|  | **peakVO_2_** | |  | **VO_2_@AT** | |  | **Oxygen pulse** | |  | **maximum power  output** | |
| --- | --- | --- | --- | --- | --- | --- | --- | --- | --- | --- | --- |
|  | **ß coefficient  (Stderr)** | **p** |  | **ß coefficient  (Stderr)** | **p** |  | **ß coefficient  (Stderr)** | **p** |  | **ß coefficient  (Stderr)** | **p** |
| ***Whole population*** |  |  |  |  |  |  |  |  |  |  |  |
| **Men (n = 286)** |  |  |  |  |  |  |  |  |  |  |  |
| adjusted for age, BMI, smoking, glucose,  total cholesterol, physical activity | -103.9 (43.225) | 0.02 |  | -9.679 (26.490) | 0.72 |  | -0.518 (0.248) | 0.04 |  | -8.077 (3.253) | 0.01 |
| **Women (n = 385)** |  |  |  |  |  |  |  |  |  |  |  |
| adjusted for age, BMI, smoking, glucose, total cholesterol, physical activity | 17.081 (22.891) | 0.46 |  | -4.734 (13.267) | 0.72 |  | -0.021 (0.148) | 0.88 |  | -2.047 (1.852) | 0.27 |
| ***Subpopulation of subjects which a time-lag between blood sample and pulmonary function testing under 1 month*** | | | | | | | | | | | |
| **Men (n = 160)** |  |  |  |  |  |  |  |  |  |  |  |
| adjusted for age, BMI, smoking, glucose, total cholesterol, physical activity | -108.7 (59.216) | 0.07 |  | 7.087 (36.147) | 0.84 |  | -0.506 (0.336) | 0.13 |  | -9.335 (4.482) | 0.04 |
| **Women (n = 204)** |  |  |  |  |  |  |  |  |  |  |  |
| adjusted for age, BMI, smoking, glucose, total cholesterol, physical activity | 69.970 (27.758) | 0.01 |  | 15.227 (18.637) | 0.41 |  | 0.400 (0.179) | 0.03 |  | 0.849 (2.293) | 0.71 |

COPD = chronic obstructive pulmonary disease; BMI = body-mass index; peakVO_2_ = highest 10-second average of VO_2_ in the last minute of exercise; VO_2_@AT = VO_2_ at anaerobic threshold. *All models were further adjusted for time between core examination and pulmonary function testing and month of core examination.

**Table S2.** Association between irisin and CPET parameters depending on different adjustment sets in a COPD free population.

|  | **peakVO_2_** | |  | **VO_2_@AT** | |  | **Oxygen pulse** | |  | **maximum power  output** | |
| --- | --- | --- | --- | --- | --- | --- | --- | --- | --- | --- | --- |
|  | **ß coefficient  (Stderr)** | **p** |  | **ß coefficient  (Stderr)** | **p** |  | **ß coefficient  (Stderr)** | **p** |  | **ß coefficient  (Stderr)** | **p** |
| ***Whole population*** |  |  |  |  |  |  |  |  |  |  |  |
| **Men (n = 286)** |  |  |  |  |  |  |  |  |  |  |  |
| adjusted for age, BMI, smoking | -101.0 (47.941) | 0.04 |  | -15.07 (28.424) | 0.60 |  | -0.449 (0.271) | 0.10 |  | -7.639 (3.449) | 0.03 |
| adjusted for age, BMI, smoking,  glucose, total cholesterol | -111.5 (48.541) | 0.02 |  | -18.09 (28.804) | 0.53 |  | -0.521 (0.274) | 0.06 |  | -8.389 (3.494) | 0.02 |
| **Women (n = 385)** |  |  |  |  |  |  |  |  |  |  |  |
| adjusted for age, BMI, smoking | 23.357 (24.401) | 0.34 |  | -0.079 (14.074) | 1.00 |  | 0.025 (0.156) | 0.87 |  | -1.034 (1.969) | 0.60 |
| adjusted for age, BMI, smoking,  glucose, total cholesterol | 19.541 (24.577) | 0.43 |  | -2.130 (14.182) | 0.88 |  | -0.015 (0.156) | 0.92 |  | -1.236 (1.986) | 0.53 |
| ***Subpopulation of subjects which a time-lag between blood sample and pulmonary function testing under 1 month*** | | | | | | | | | | | |
| **Men (n = 160)** |  |  |  |  |  |  |  |  |  |  |  |
| adjusted for age, BMI, smoking | -130.2 (65.613) | 0.05 |  | 6.661 (40.302) | 0.87 |  | -0.535 (0.374) | 0.16 |  | -9.828 (4.835) | 0.04 |
| adjusted for age, BMI, smoking,  glucose, total cholesterol | -103.1 (63.885) | 0.11 |  | 7.871 (38.879) | 0.84 |  | -0.387 (0.367) | 0.29 |  | -8.073 (4.693) | 0.09 |
| **Women (n = 204)** |  |  |  |  |  |  |  |  |  |  |  |
| adjusted for age, BMI, smoking | 73.152 (29.707) | 0.01 |  | 20.547 (20.367) | 0.31 |  | 0.403 (0.189) | 0.03 |  | 1.689 (2.473) | 0.50 |
| adjusted for age, BMI, smoking,  glucose, total cholesterol | 72.904 (29.450) | 0.01 |  | 22.428 (20.183) | 0.27 |  | 0.414 (0.187) | 0.03 |  | 1.800 (2.449) | 0.46 |

COPD = chronic obstructive pulmonary disease; BMI = body-mass index; peakVO_2_ = highest 10-second average of VO_2_ in the last minute of exercise; VO_2_@AT = VO_2_ at anaerobic threshold. *All models were further adjusted for time between core examination and pulmonary function testing and month of core examination.
